# Breast Cancer–Protective Genetic Variant Common in Women of Indigenous American Genetic Ancestry Is a cis-eQTL for *RMND1*, *ARMT1*, and *CCDC170* in HER2-Enriched Tumor Subtypes

**DOI:** 10.1158/2767-9764.CRC-26-0293

**Published:** 2026-10-09

**Authors:** Valentina A. Zavala, Xiaosong Huang, Sandro Casavilca-Zambrano, Jeannie Navarro-Vásquez, Carlos A. Castañeda, Guillermo Valencia, Zaida Morante, Monica Calderón, Julio E. Abugattas, Henry Gómez, Hugo A. Fuentes, Ruddy Liendo-Picoaga, Jose M. Cotrina, Claudia Monge-Pimentel, Silvia P. Neciosup, Jule Vásquez, Marco Gálvez-Niño, Víctor Castro, Luis A. Salinas Agramonte, Patricia Rioja Viera, Mileine Bravo-Poemape, Luis Mas, Scott Huntsman, Donglei Hu, Jovanny Zabaleta, Elad Ziv, Tatiana Vidaurre, Laura Fejerman

**Affiliations:** 1 Department of Public Health Sciences, University of California, Davis, Davis, California.; 2 Instituto Nacional de Enfermedades Neoplasicas, Lima, Peru.; 3 Division of General Internal Medicine, Department of Medicine, University of California, San Francisco, San Francisco, California.; 4 Department of Interdisciplinary Oncology, Louisiana State University Health Sciences Center, New Orleans, Louisiana.; 5 UC Davis Comprehensive Cancer Center, University of California, Davis, Davis, California.

## Abstract

Breast cancer genome-wide association studies in Hispanic/Latina women in the United States and Latin America have identified independent risk variants in the 6q25 region, including the protective variant rs140068132, located upstream of the estrogen receptor 1 (*ESR1*) gene. The molecular mechanisms linking this and other variants to breast cancer risk remain poorly understood. To investigate the potential functional role of rs140068132, we analyzed the association between this variant and gene expression in breast cancer tissue. We performed RNA sequencing on 270 tumors from the Peruvian Genomics of Breast Cancer Study and determined intrinsic tumor subtypes using PAM50. The effect of rs140068132 on gene expression across tumor subtypes was assessed using models adjusted for age at diagnosis, Indigenous American genetic ancestry, PAM50 subtype, and an interaction term between tumor subtype and SNP genotype. Subtype-specific effects of the protective rs140068132-G allele were observed in HER2-enriched tumors, with increased expression of *ARMT1*, *RMND1*, and *CCDC170*. These results suggest that in breast cancer tissue from Peruvian patients, rs140068132 influences the expression of multiple genes in a subtype-specific manner, likely acting through a regulatory element at 6q25 that modulates gene expression in cis depending on cellular context.

**Significance:**

A protective germline variant, common in women of Indigenous American ancestry, upregulates the expression of cis-genes only in HER2-enriched tumors, providing valuable information for model selection in downstream mechanistic studies.

## Introduction

The identification of genetic variants associated with breast cancer susceptibility and progression has been a critical focus of research, enabling a better understanding of the underlying molecular mechanisms and facilitating personalized prevention and treatment strategies (1). Genome-wide association studies (GWAS) have played a pivotal role in identifying hundreds of genetic variants associated with breast cancer risk; however, most of these variants have been discovered in women of European and Asian genetic ancestry (2). Even though the inclusion of diverse populations has increased over the last 20 years, US Hispanic/Latinos continue to be underrepresented in GWAS and transcriptome databases (3). Lack of representation and small sample size, together with different patterns of linkage disequilibrium, can explain why some index risk polymorphisms reported for other populations did not show strong associations in previous studies of US Hispanic/Latino and Latin American patients (4–6).

In this article, we use the constructs of race and ethnicity to refer to what we know are heterogeneous groups of people defined by the US Office of Management and Budget as African Americans/Blacks, American Indians and Alaska Natives, Asians, Native Hawaiians/other Pacific Islanders, Non-Hispanic/Latino White, and Hispanics/Latinos (7). These social constructs are different from the concept of genetic ancestry, which is estimated based on genome-wide genotype data, leveraging allele frequency differences between continental and subcontinental groups due to human demographic histories (8). The term Indigenous American genetic ancestry refers to the component of genetic ancestry that is similar to that of populations that were in the American continent before the European colonization often referred to as Native American genetic ancestry (8).

The largest breast cancer GWAS conducted in US Hispanic/Latina (H/L) and Latin American women has consistently identified the rs140068132 SNP in the 6q25 region as the strongest signal (4–6). The frequency of the minor allele is correlated with Indigenous American genetic ancestry and is associated with lower odds of developing breast cancer, with an estimate of ∼0 minor allele frequency in populations of European and African descent without Indigenous American genetic ancestry (4, 9). The finding of this protective variant is concordant with the lower incidence of breast cancer in US H/L and Latin American women compared with non-H/L White women (10). Besides rs140068132, the 6q25 locus contains multiple polymorphisms associated with breast cancer risk described in populations of European and Asian genetic ancestry (11–18) and associated with subtype-specific effects (11, 12, 15). We have identified SNPs in the 6q25 region in H/L and Latin American individuals who retained their significance in conditional models accounting for rs140068132, suggesting their independent contribution to breast cancer susceptibility (6). In addition, we reported that the rs140068132 polymorphism was not only associated with a decreased risk of breast cancer but was also more protective against hormone receptor–negative (HR−) and HER2+ diseases, independently of other known breast cancer–associated variants in the 6q25 locus (6), suggesting effect heterogeneity by subtype for this region.

Several studies have defined independent breast cancer risk variants in the 6q25 as expression quantitative trait loci (eQTL) for estrogen receptor 1 gene (*ESR1*) and other genes in the region, such as required for meiotic nuclear division 1 homolog (*RMND1*), acidic residue methyltransferase 1 (*ARMT1*), and coiled-coil domain containing 170 (*CCDC170*; refs. 17, 19–23). Variants within the *CCDC170*, *RMND1*, and *ESR1* genes have been associated with a risk of osteoporosis and bone mineral density (24), both phenotypes associated with estrogen levels (25), supporting the potential regulatory effects of variants in this region on estrogen receptor (ER)–dependent pathways. Prior evidence suggests a potential functional role of the rs140068132 polymorphism as it lies within a transcription factor–binding site (56 kilobases upstream of the *ESR1* gene) and disrupts binding based on an electrophoretic mobility shift assay (4). However, the absence of Hispanic/Latino representation in databases with both germline and tissue transcriptomic characterization has limited progress in the investigation of the functional implications of this and other population-specific variants in the 6q25 region. Given the evidence linking rs140068132 with functionality, we investigated its association with gene expression in breast cancer tissue from a subset of Peruvian women with breast cancer to further understand the possible mechanisms linking polymorphisms within the 6q25 region and subtype-specific breast cancer risk in US H/L and Latin American individuals.

## Materials and Methods

### Study participants

*The Peruvian Genetics and Genomics of Breast Cancer Study* (PEGEN-BC): As of October 2025, 2,156 participants were recruited from the Instituto Nacional de Enfermedades Neoplásicas (INEN) in Lima, Peru, the largest cancer hospital in the country. A description of the cohort has been published elsewhere (26). Briefly, women were invited to participate if they had a diagnosis of invasive breast cancer in the year 2010 or later and were between 21 and 79 years of age when diagnosed. Demographic and clinical data were abstracted from electronic medical records (27), and a blood sample was drawn by a certified phlebotomist at the INEN central laboratory. The current analysis includes a subset of 270 samples from a set of 500 patients with available tumor tissue. These tumors were selected to represent the different subtypes and included as many participants with the rs140068132-G allele as possible.

Patient and tumor characteristics of individuals in the PEGEN-BC study are similar to those previously described for the overall INEN breast cancer population (28). This study was approved by the INEN and the University of California Davis Institutional Review Boards.

### Continental genetic ancestry estimation

Although US Hispanic/Latino is a US Census Bureau ethnic category that may include individuals of any racial identity or genetic background, the following analyses focus on continental genetic ancestry, which is estimated from genomic data. Estimation of continental genetic ancestry was performed on genome-wide genotype data as previously described (6). Briefly, genotype data were obtained with the Affymetrix Precision Medicine Research Array for the PEGEN-BC study participants. Quality control (QC) and pruning were performed using PLINK v.1.9 (RRID: SCR_001757; window size = 50, number of variants = 5, and variance inflation factor threshold = 2; ref. 29), and data were merged with data from the 1,000 Genomes Project (RRID: SCR_006828; ref. 30), including Admixed Americans (Peru, Colombia, Mexico, and Puerto Rico), Europeans (Americans with Northern and Western European Ancestry, Italy, Spain, Finland, and Scotland), East Asians (China, Japan, and Vietnam), and African populations (Nigeria, Kenya, The Gambia, and Sierra Leone). The PEGEN-BC study includes a large proportion of patients with >98% Indigenous American genetic ancestry, as previously reported (26, 27), and therefore provides a source of reference samples for this component. Individual global genetic ancestry was estimated using ADMIXTURE (RRID: SCR_001263; unsupervised, k = 4; ref. 31).

### RNA sequencing and data processing

Tumor tissues were obtained from core biopsy or freshly resected invasive breast cancers before treatment that were formalin-fixed and paraffin-embedded (FFPE) following standard protocols at INEN. Total RNA was extracted from 290 FFPE breast tumor tissue samples from PEGEN-BC patients. RNA extraction was performed using the AllPrep DNA/RNA FFPE Kit (Qiagen) following the protocol provided by the manufacturer. RNA quality and quantity were assessed. Samples with DV200 >30% were selected for library preparation. DV200 is a measure of the percentage of RNA fragments with a length >200 nucleotides (32) and has been shown to be a reliable measure of quality (33). Libraries were prepared using the Illumina RNA Exome Kit, quantified, and pooled. Two rounds of hybridization with probes were carried out and captured libraries were cleaned up, enriched, and amplified. Resulting libraries were quantified, and QC was performed using Agilent 2100 BioAnalyzer (Agilent Technologies, RRID: SCR_018043). Paired-end exome sequencing (2 × 75 bp paired-end, 100 million reads per sample) was performed in an Illumina NextSeq 500 High Output Flow Cell Kit v2.5 (Illumina Inc.). Starting from paired-end RNA sequencing data in FASTQ format, adapters were trimmed off and low-quality reads were removed using fastp (34) v.0.19.6’s default settings (RRID: SCR_016962).

Reads were aligned against an rRNA reference using bwa-mem (RRID: SCR_010910; ref. 35) to obtain transcript counts at the whole-gene and -isoform levels. With low-quality reads and putative rRNA reads removed, the remaining sequences were aligned against the hg38 reference using STAR (36) v2.6.0 (RRID: SCR_004463), and expression was calculated using RSEM (RRID: SCR_000262; ref. 37). Duplicates were marked using GATK’s MarkDuplicates (RRID: SCR_001876; ref. 38), and QC was finally compiled from a combination of fastp’s summaries of reads found in the originating FASTQ files, samtools (RRID: SCR_002105; ref. 38) flagstat counts in aligned bam, and Picard’s CollectRnaSeqMetrics and CollectAlignmentSummaryMetrics (RRID: SCR_006525; ref. 39).

### Breast cancer tumor subtype classification

Tumor-intrinsic subtypes were determined using an in-house adaptation of the PAM50 method to account for the different proportions of tumors based on ER status in our cohort compared with those from the dataset from the PAM50 publication (40). Prior to classification, we sampled a subgroup of the training cohort with a clinicopathologic distribution similar to that of the study cohort. For this subgroup, we found the expression value at the percentile “Q_gene” that corresponded to the percentile of the baseline expression “mu_gene” of the entire training cohort, hence estimating where the baseline expression “mu_gene” of the entire training cohort laid within the study cohort. We then assigned the baseline expression of each gene at the “Q_gene” percentile and centered the gene expression by subtracting the value at this percentile. The PAM50 method was executed using the genefu package in R (41). Tumors classified as normal-like (*n* = 7) were removed from the analysis as this class was represented using tissue from reduction mammoplasty or grossly uninvolved tissue (40); therefore, they reflect a gene expression pattern similar to normal breast tissue.

### Statistical analyses

*Differential gene expression analysis* was performed using the DESeq2 R package (RRID: SCR_015687; ref. 42), adjusting by age at diagnosis and Indigenous American genetic ancestry and including PAM50 subtype or an interaction term between subtype and the rs140068132 genotype, with HER2-enriched tumors as the reference group to assess its effect across subtypes and subtype-specific effects, respectively. Samples with stage IV cancer (*n* = 11) and patients carrying the rs140068132-G/G genotype (*n* = 2) were excluded from the analysis because of their low frequency among the sequenced tumor samples, leaving a total of 270 samples for downstream analysis (Table 1). All genes (*n* = 28,278) were included in the analysis to ensure proper normalization (library size correction) of the data and that the priors were well constructed from the dispersion log_2_ fold change estimation steps. We extracted results of genes within the 1Mb window around the rs140068132 SNP.

**Table 1.** Anthropometric, clinical, and genetic characteristics of patients with breast cancer included in the study, presented overall and stratified by molecular subtype.

Characteristic	Overall	Luminal A	Luminal B	HER2-enriched	Basal-like	*P* value
*n* (%)	270 (100)	54 (20)	83 (30.7)	73 (27)	60 (22.2)	​
Age, years [mean (SD)]	50.76 (10.42)	51.50 (11.09)	51.75 (10.09)	51.03 (10.18)	48.40 (10.43)	0.247
BMI, kg/m^2 ^[mean (SD)]	27.94 (4.62)	28.41 (4.41)	28.33 (4.88)	27.82 (4.36)	27.10 (4.72)	0.370
Height, cm [mean (SD)]	153.21 (6.70)	154.79 (7.86)	152.87 (6.53)	151.90 (6.41)	153.83 (5.91)	0.094
Stage, *n* (%)	​	​	​	​	​	​
I	6 (2.2)	3 (5.6)	2 (2.4)	0 (0)	1 (1.7)	0.001
II	112 (41.5)	34 (63)	37 (44.6)	22 (30.1)	19 (31.7)	​
III	152 (56.3)	17 (31.5)	44 (53)	51 (69.9)	40 (66.7)	​
Indigenous American genetic ancestry, mean (SD)	0.77 (0.16)	0.76 (0.18)	0.78 (0.16)	0.80 (0.14)	0.76 (0.17)	0.470
rs140068132 genotype, *n* (%)	​	​	​	​	​	​
AA	202 (74.8)	37 (68.5)	57 (68.7)	57 (78.1)	51 (85)	0.183
AG	66 (24.4)	16 (29.6)	26 (31.3)	15 (20.5)	9 (15)	​
GG	2 (0.7)	1 (1.9)	0 (0)	1 (1.4)	0 (0)	​

Abbreviation: BMI, body mass index.

Patients with stage IV disease (*n* = 11) and those classified as normal-like PAM50 (*n* = 7) were excluded from the analysis and are therefore not shown. Categorical variables were compared using the *χ*^2^ test, and continuous variables were analyzed using one-way ANOVA.

*P* values were adjusted for multiple testing across all genes using the Benjamini–Hochberg procedure to control the FDR, and genes with FDR-adjusted *P* values < 0.05 were considered statistically significant. R v.3.6.0 was used for data management, analyses, and visualization (43).

Pairwise correlation plots were generated using the GGally::ggpairs function in R (RRID: SCR_026114; ref. 44), which generates scatterplots, density plots, and Pearson correlation coefficients for all variable pairs.

## Results

We previously reported that the rs140068132 polymorphism is not only associated with a decreased risk of breast cancer but is also more protective against HR− and HER2+ subtypes. This association is independent of other known breast cancer–associated variants in the 6q25 locus: rs851984, rs9918437, rs2228480, and rs3798758 (6). Here, we aimed to assess the impact of rs140068132 on gene expression in cis to explore its potential role as breast cancer subtype-specific eQTL.

Among patients whose tumors were sequenced and included in this study (*n* = 270), the average age at diagnosis was 50.8 years (SD = 10.4), and the mean Indigenous American genetic ancestry was 77% (SD = 16%). According to the PAM50 classification, 54 tumors were luminal A, 83 luminal B, 73 HER2-enriched, and 60 basal-like (Table 1).

### Association between rs140068132 genotype and cis gene expression

According to the GENCODE Genes track in the UCSC database, the 6q25 region contained 103 genes (Known Genes track). Although there is no fixed limit, cis-eQTLs are typically defined as variants located within 1 to 5 Mb from the gene of interest (eGene; ref. 45). Given the limited power of our study to detect more distant eQTLs (including tras-eQTLs) due to sample size, we defined a 1-Mb window around the rs140068132 SNP as an appropriate region to explore its effect as a cis-eQTL (chr6:150,633,599–152,633,799; hg38).

**Figure 1.**
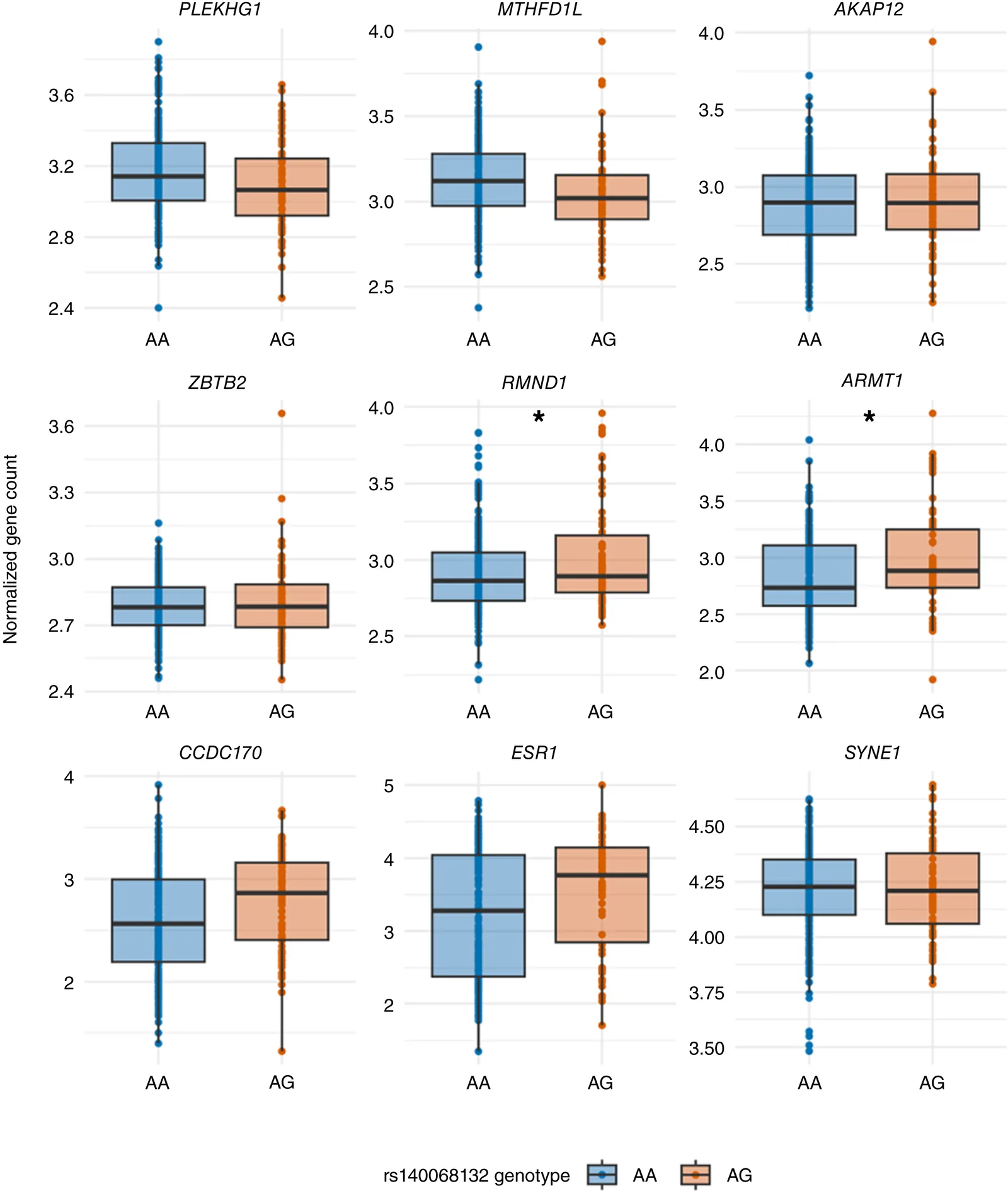
Differential gene expression by rs140068132 genotype. Genes within ±1 Mb of rs140068132 are displayed in genomic order. Statistically significant associations after FDR correction in analyses adjusted by age, Indigenous American genetic ancestry, and PAM50 subtype are indicated with an asterisk.

Among the nine genes located within ±1 Mb of rs140068132, *RMND1* and *ARMT1* exhibit statistically significant differential expression levels by rs140068132 allele (Fig. 1). To identify cis-eGenes potentially involved in the mechanisms through which rs140068132 confers protection against breast cancer, we conducted linear regression analyses adjusted for age at diagnosis and Indigenous American genetic ancestry, with additional models including PAM50 tumor subtype to evaluate whether subtype acted as a potential confounder. In both modeling approaches, among the nine protein-coding genes within this region, *ARMT1* and *RMND1* demonstrated statistically significant differential expression after FDR correction (Table 2), whereas *CCDC170* expression showed a borderline association. Additional adjustment for tumor stage did not change the results; effect directions and statistical significance were consistent with the primary analyses, with the exception of *CCDC170*, which became statistically significant after FDR correction (Table 2). As adjustment for stage did not meaningfully improve the model, we did not include it in subsequent analyses, particularly given the limited number of stage I tumors (Table 1).

**Table 2.** Differential gene expression of genes within a 1-Mb window from rs140068132 in model adjusted by age and Indigenous American genetic ancestry, with and without adjustment for PAM50 breast cancer subtype.

Gene	Log_2_ FC	lfcSE	Wald statistic	*P* value	*P* adjusted
Model adjusted by age, IAA					
*AKAP12*	0.18	0.13	1.36	0.173	0.494
*ARMT1*	0.94	0.18	5.18	<0.001	**<0.001**
*CCDC170*	0.61	0.21	2.9	<0.001	0.073
*ESR1*	0.53	0.3	1.77	0.078	0.339
*MTHFD1L*	−0.22	0.11	−1.89	0.059	0.297
*PLEKHG1*	−0.29	0.12	−2.47	0.014	0.143
*RMND1*	0.63	0.15	4.31	<0.001	**0.003**
*SYNE1*	0.04	0.09	0.46	0.642	0.87
*ZBTB2*	0.17	0.07	2.33	0.02	0.174
Model adjusted by age, IAA, and tumor stage					
*AKAP12*	0.16	0.12	1.28	0.202	0.510
*ARMT1*	0.94	0.17	5.49	<0.001	**<0.001**
*CCDC170*	0.83	0.19	4.33	<0.001	0.002
*ESR1*	0.68	0.28	2.38	0.017	0.150
*MTHFD1L*	−0.26	0.11	−2.41	0.016	0.144
*PLEKHG1*	−0.29	0.11	−2.60	0.009	0.107
*RMND1*	0.59	0.14	4.29	<0.001	0.003
*SYNE1*	0	0.09	0.03	0.976	0.995
*ZBTB2*	0.10	0.07	1.48	0.139	0.426
Model adjusted by age, IAA, and PAM50 subtype					
*AKAP12*	0.15	0.13	1.16	0.245	0.724
*ARMT1*	0.56	0.13	4.36	<0.001	**0.005**
*CCDC170*	0.42	0.16	2.71	0.007	0.19
*ESR1*	0.19	0.19	0.97	0.33	0.792
*MTHFD1L*	−0.06	0.09	−0.68	0.495	0.883
*PLEKHG1*	−0.06	0.09	−0.64	0.519	0.893
*RMND1*	0.43	0.12	3.59	<0.001	**0.047**
*SYNE1*	0.01	0.09	0.07	0.947	0.996
*ZBTB2*	0.15	0.07	2.02	0.044	0.411

Abbreviations: FC, fold change; IAA, Indigenous American Ancestry; lfcSE, log_2_ fold change standard error.

Log_2_ fold change: estimated effect size. Positive value indicates upregulation, whereas a negative value indicates downregulation in rs140068132-G carriers compared with that in noncarriers. Log_2_ fold change standard error: standard error estimate for the log_2_ fold change. Wald statistic: test statistic calculated as the log_2_ fold change divided by the lfcSE. *P* value: unadjusted *P* value associated with the Wald statistic. *P* adjusted: FDR-adjusted *P* value for multiple testing across all genes, calculated using the Benjamini–Hochberg procedure. *P* adjusted values <0.05 were considered statistically significant and are shown in bold.

### Association between rs140068132 genotype and cis gene expression stratified by tumor subtype

**Figure 2.**
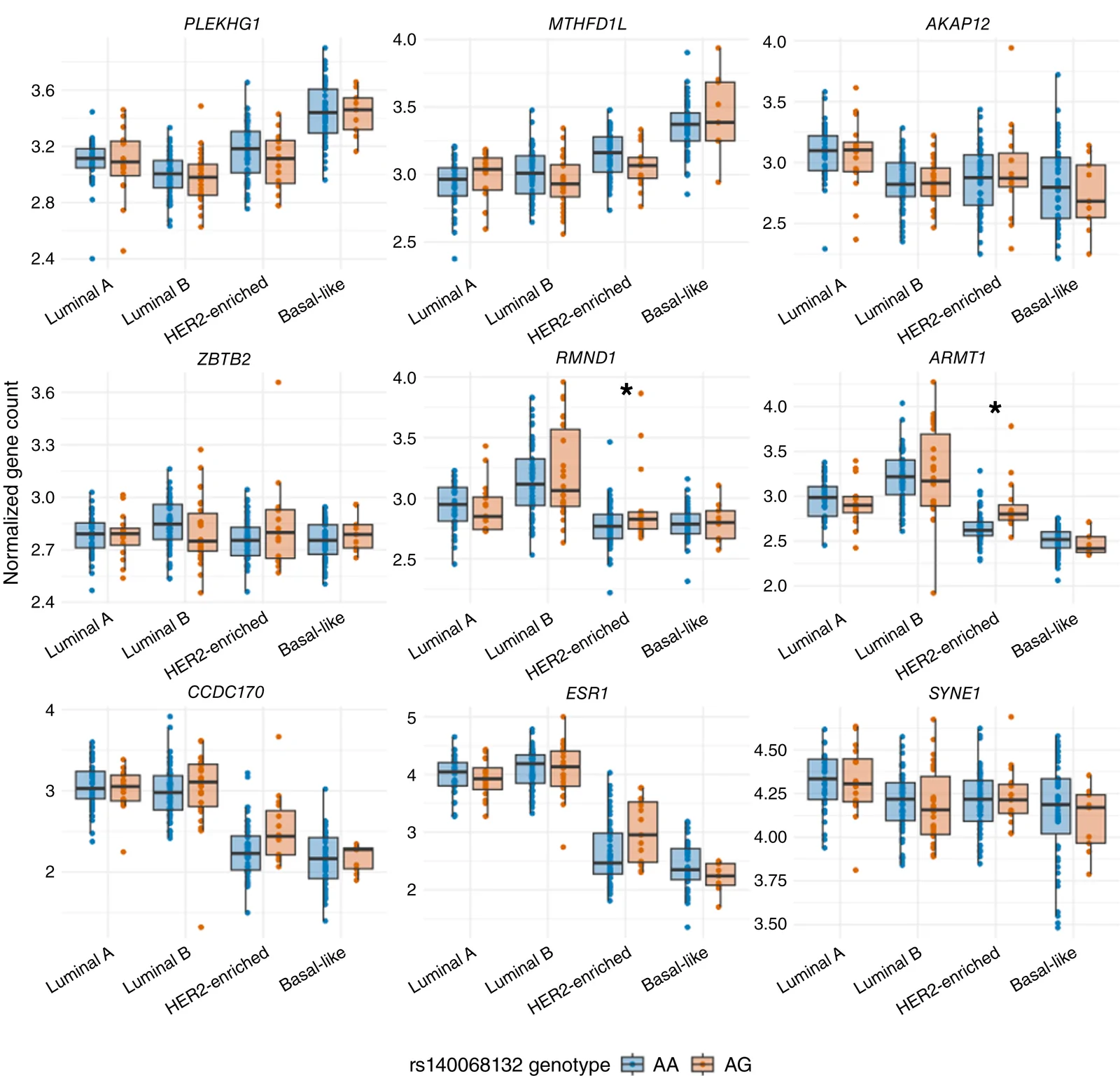
Differential gene expression by rs140068132 genotype by tumor subtype. Genes within ±1 Mb of rs140068132 are displayed in genomic order. Statistically significant associations after FDR correction in analyses stratified by tumor subtype are indicated with an asterisk.

Given the subtype-specific association between rs140068132 and breast cancer risk, and the substantial transcriptomic heterogeneity captured by PAM50 classification, we further examined this relationship using a PAM50 subtype–stratified analysis. The genotype distribution across tumor subtypes is described in Table 1. *ARMT1*, *CCDC170*, and *RMND1* showed statistically significant associations after FDR correction specifically within the HER2-enriched subtype, whereas no additional genes demonstrated evidence of subtype-restricted effects (Table 3; Fig. 2). Although not statistically significant after FDR correction, *ESR1* expression tended to be higher among G-allele carriers in tumors from patients with the HER2-enriched subtype. This HER2-enriched–specific effect explained the trend toward higher *ESR1* levels observed in the analysis including all tumor subtypes (Table 2).

**Table 3.** Differential gene expression of genes within a 1-Mb window from rs140068132 in model adjusted by age and Indigenous American genetic ancestry, stratified by PAM50 breast cancer subtype.

Gene	Log_2_ FC	lfcSE	Wald statistic	*P* value	*P* adjusted
Luminal A					
*AKAP12*	0.05	0.24	0.21	0.830	0.999
*ARMT1*	−0.16	0.22	−0.74	0.457	0.999
*CCDC170*	−0.23	0.26	−0.88	0.381	0.999
*ESR1*	−0.18	0.29	−0.62	0.533	0.999
*MTHFD1L*	0.11	0.18	0.62	0.536	0.999
*PLEKHG1*	0.08	0.18	0.45	0.655	0.999
*RMND1*	−0.03	0.2	−0.14	0.891	0.999
*SYNE1*	0.11	0.17	0.64	0.525	0.999
*ZBTB2*	0.01	0.12	0.1	0.921	0.999
Luminal B					
*AKAP12*	0.04	0.16	0.24	0.813	0.987
*ARMT1*	0.81	0.27	3.05	0.002	0.175
*CCDC170*	0.38	0.24	1.54	0.123	0.653
*ESR1*	0.24	0.25	0.96	0.339	0.837
*MTHFD1L*	−0.21	0.15	−1.46	0.145	0.677
*PLEKHG1*	−0.02	0.13	−0.15	0.883	0.996
*RMND1*	0.48	0.26	1.87	0.062	0.538
*SYNE1*	0.01	0.15	0.09	0.931	0.997
*ZBTB2*	−0.07	0.13	−0.54	0.589	0.938
HER2-enriched					
*AKAP12*	0.67	0.28	2.44	0.015	0.239
*ARMT1*	1.02	0.22	4.62	<0.001	**0.002** ^a^
*CCDC170*	1.15	0.32	3.56	<0.001	**0.038** ^a^
*ESR1*	0.73	0.47	1.56	0.120	0.544
*MTHFD1L*	−0.36	0.15	−2.34	0.019	0.27
*PLEKHG1*	−0.27	0.19	−1.4	0.161	0.605
*RMND1*	0.92	0.23	3.93	<0.001	**0.017** ^a^
*SYNE1*	0.08	0.17	0.45	0.650	0.927
*ZBTB2*	0.54	0.17	3.14	0.002	0.086
Basal-like					
*AKAP12*	−0.34	0.39	−0.86	0.392	0.999
*ARMT1*	−0.05	0.18	−0.29	0.775	0.999
*CCDC170*	−0.27	0.37	−0.74	0.458	0.999
*ESR1*	−0.92	0.43	−2.16	0.031	0.879
*MTHFD1L*	0.41	0.26	1.62	0.105	0.999
*PLEKHG1*	−0.07	0.25	−0.28	0.781	0.999
*RMND1*	0.11	0.17	0.62	0.534	0.999
*SYNE1*	−0.2	0.29	−0.69	0.489	0.999
*ZBTB2*	0.13	0.14	0.94	0.349	0.999

Abbreviations: FC, fold change; lfcSE, log_2_ fold change standard error.

a Indicates a statistically significant (*P* < 0.05) interaction between subtype and rs140068132 genotype (interaction test results are shown in Supplementary Table S1). Log_2_ fold change: estimated effect size. Log_2_ fold change standard error: standard error estimate for the log_2_ fold change. Wald statistic: test statistic calculated as the log_2_ fold change divided by the log_2_ fold change standard error. *P* value: unadjusted *P* value associated with the Wald statistic. *P* adjusted: FDR-adjusted *P* value for multiple testing across all genes, calculated using the Benjamini–Hochberg procedure. *P* adjusted values <0.05 were considered statistically significant and are shown in bold.

To assess whether the effects observed on gene expression were specific to rs140068132, we performed additional analyses adjusting for rs9918437. rs9918437 is a breast cancer risk variant described in European and Asian GWASs and a known eQTL for genes in the 6q25 region (11). We have previously reported that, unlike the other known breast cancer risk variants (6), it is associated with increased odds of HR−/HER2+ disease (based on IHC classification) in the PEGEN-BC cohort (6). We observed that this variant was not associated with gene expression in the 6q25 region and that the associations observed for rs140068132 remained significant after including rs9918437 in the model (Supplementary Table S2).

We assessed heterogeneity in the association between rs140068132 genotype and gene expression across PAM50 subtypes using a genotype–subtype interaction model adjusted for age at diagnosis and Indigenous American genetic ancestry. The HER2-enriched subtype was defined as the reference group. After adjustment for multiple testing, significant interaction effects were observed only for *ARMT1*, *CCDC170*, and *RMND1*, indicating that these associations were specific to the HER2-enriched subtype. No genes showed significant interaction effects in any of the other PAM50 subtypes relative to the HER2-enriched reference group (Supplementary Table S1).

**Figure 3.**
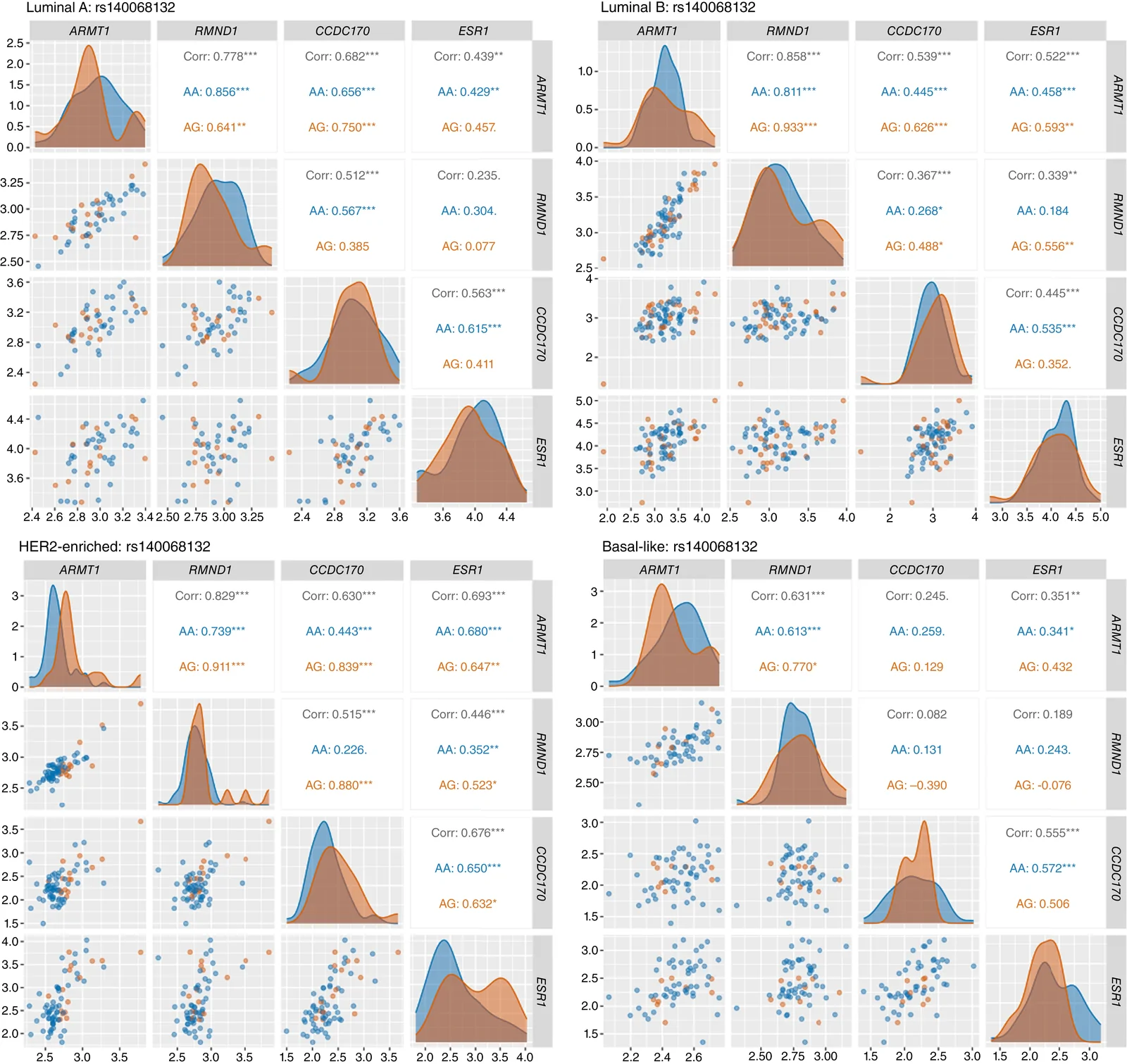
Generalized pairs plot illustrating pairwise correlations among genes significantly associated with the rs140068132 genotype and *ESR1*, stratified by PAM50 tumor subtype. All values are calculated using log_10_-transformed raw data. Values labeled “Corr” represent the overall Pearson correlation between each gene pair across all samples, whereas values labeled AA and AG indicate correlations calculated separately within noncarriers (AA) and carriers (AG) of the rs140068132 variant. *, *P* < 0.05; **, *P* < 0.01; ***, *P* < 0.001.

Scatterplots stratified by PAM50 subtype were used to analyze the distribution of expression levels of the genes found statistically significantly associated with rs140068132 genotype: *ARMT1*, *CCDC170*, and *RMND1*. Despite the absence of a statistically significant association with rs140068132 genotype, *ESR1* was included given its biological relevance in breast cancer development and the reported co-expression in breast tissue with CCDC170 and RMND1, providing relevant biological context. We observed that the effect of the rs140068132-G allele was most pronounced in the HER2-enriched subtype. Within this group, pairwise correlations were consistently higher among carriers compared with noncarriers, suggesting stronger coregulation of these genes in the presence of the rs140068132-G allele (Fig. 3; Supplementary Fig. S1).

## Discussion

The effect of breast cancer–associated genetic variants is mainly exerted through the disruption of regulatory regions and/or splicing (46, 47), which affects the expression of genes and molecular pathways (11, 21, 22, 48–50). These changes can lead to the development of specific breast cancer phenotypes (11, 12, 15) and consequently, to different prognosis and survival (48, 49, 51, 52). The present study aimed to assess the effect of rs140068132, a known breast cancer–protective variant in the 6q25 locus (4–6), on the cis regulation of genes in breast cancer tumors from women in Peru. Previous studies have shown that breast cancer–associated SNPs at the 6q25 locus influence the expression of a coregulated gene cluster including *ARMT1*, *RMND1*, *CCDC170*, and *ESR1* (53). Using multiple regression modeling approaches adjusted by subtype, we found that rs140068132 was associated with the expression of *ARMT1* and *RMND1*. Consistent with the subtype-specific effect of this SNP, which decreases risk for the HR− HER2+ subtype, the variant showed stronger effects on the expression of *ARMT1*, *CCDC170*, and *RMND1* in HER2-enriched tumors. This subtype-specific association could be explained by distinct tumorigenic pathways operating in different breast cancer subtypes, although we acknowledge that limited statistical power may also contribute to the lack of evidence for such associations in other PAM50 subtypes.

ARMT1 is a protein-glutamate O-methyltransferase implicated in breast cancer cell survival, suggesting that its upregulation could modulate tumor growth in a subtype-specific context (54). *RMND1* encodes a protein that localizes to the inner mitochondrial membrane, but its precise functional role has not yet been fully characterized. Although no evidence links *RMND1* and HER2-related pathways, HER2 protein, which is mainly localized in the cell membrane, can also translocate to mitochondria and promote tumor growth and metastasis, independently from the HER2 membrane signaling pathways (55). The CCDC170 protein promotes bone formation, and deleterious variants have been shown to reduce bone mineral density, increase the risk of osteoporosis, and associate with reduced breast cancer risk (56, 57). ΔCCDC170 fusion proteins can modulate HER2/HER3 signaling and endocrine responsiveness, highlighting a potential mechanistic link to the biology of HER2-enriched tumors (58) and have been identified in up to 4% or ER+ tumors (59). It is important to note, however, that the association between *CCDC170* expression and rs140068132 genotype observed in this study was borderline and subtype-specific and should therefore be interpreted with caution until confirmed in independent replication studies.

We have previously reported that the rs140068132-G allele, which correlates with Indigenous American genetic ancestry, is associated with a lower risk of developing HR− and HER2+ breast cancer subtypes, compared with HR+/HER2− disease (6). This polymorphism is located 56 kb upstream of the *ESR1* gene, within a known regulatory region, and is thought to disrupt an enhancer region by reducing the binding affinity of several transcription factors (4). Our results suggest that this variant affects gene expression of several genes in *cis*, consistent with its likelihood of affecting a cis-regulatory element.

Genes within the 6q25.1 locus reside in hormone-responsive regions, in which exposure to estradiol and progestins alters intrachromatin interactions, leading to increased transcriptional activity (60). However, there is a lack of evidence with regard to the direct effect of these polymorphisms on susceptibility to hormonal regulation. Along these lines, we observed only a trend toward increased *ESR1* expression in HER2-enriched tumors. This might reflect fine posttranscriptional regulation of *ESR1* mRNA (61–63), which could differ in the context of a HER2+ tissue. In addition, our results reflect SNP–gene associations in the context of bulk tumor tissue, whereas the variant may affect *ESR1* expression in a specific subset of cells critical for breast cancer development, which would not be detectable in bulk analyses. Finally, another possible explanation for the lack of association between rs140068132 and *ESR1* levels is that the variant may have an effect on *ESR1* expression at different stages of tumor development, such as in premalignant stages, in a way that is not reflected in invasive breast cancer. *In vitro* experiments may help to delineate the potential regulatory role of rs140068132 on *ESR1* expression in different breast normal and cancer cells to elucidate whether regulation of *ESR1* can occur in normal tissue and/or early stages of cancer development. A small sample of propagated normal breast cells from US H/L women carrying the rs140068132-G allele has shown enhanced differentiation capacity and higher levels of luminal cell markers compared with cells generated from H/L women not carrying this protective allele (64). Breast tissues from women of high Indigenous American genetic ancestry were enriched for cells with alveolar progenitor state whereas those from women of African genetic ancestry showed enrichment of stromal fibroblasts. These cells express higher levels of *ESR1* (64), which could be partially because of the observed genetic ancestry–dependent variability in chromatin accessibility. These observations suggest that there could be additional genetic ancestry–specific determinants on the stability and/or transcription efficiency of *ESR1* beyond germline genetic susceptibility.

Our study presents some limitations. The association between genes of interest and rs140068132 genotype across PAM50 subtypes remains explorative because of the small sample size and genotype distribution (66 heterozygous and 2 homozygous carriers, with only 9 rs140068132-AG carriers among basal-like), which limits statistical power to detect subtype-specific effects. Furthermore, this variant is currently underrepresented in publicly available databases, including population-level resources such as 1,000 Genomes, Human Genome Diversity Project, and genome aggregation database, as well as phenotype-based databases such as The Genotype-Tissue Expression project, as it has only been identified in US Hispano/Latino individuals and Latin Americans, restricting broader cross-database analyses. Future studies with greater inclusion of rs140068132 carriers across subtypes will help elucidate the subtype-specific effect of this variant on *cis*- and *trans*-regulated genes, as well as pathway-level effects. Meanwhile, ongoing *in vitro* experiments using CRISPR technology to functionally characterize the rs140068132 variant will provide evidence for the underlying molecular mechanisms of breast tumor development.

Despite these limitations, our findings represent a valuable contribution to the field. By identifying rs140068132 as a variant with heterogeneous association with gene expression by subtype in an underrepresented population, this study highlights the importance of including genetically diverse individuals in cancer genomics research. These associations, once validated, could contribute to the development of preventative treatments as well as new treatment strategies for patients with breast cancer.

In conclusion, although functional causality between rs140068132 and the regulation of *ESR1*, *ARMT1*, *CCDC170*, and *RMND1* has not been established, our results are consistent with the possibility that this variant is associated with regulatory effects on breast cancer–related pathways, particularly in the context of HER2-enriched tumors. The observed subtype-specific associations may also be influenced by genetic ancestry, which has been reported to contribute to interindividual variability in chromatin organization and accessibility, potentially affecting transcriptional regulation. Although these genes are not known to be part of canonical HER2 signaling pathways, their associations with rs140068132 in HER2-enriched tumors support a context-dependent regulatory relationship that requires further investigation. Overall, these findings highlight the variant’s potential as a cis-regulatory element and suggest that its protective effects might be functionally heterogeneous depending on molecular subtype, providing valuable information for model selection in downstream mechanistic studies.

## Supplementary Material

Supplementary Table S1 Table S1. Coefficients and associated p-values for the interaction factor in a genotype–subtype interaction model evaluating the association between rs140068132 genotype and gene expression within a 1Mb window from rs140068132.

Supplementary Table S2 Table S2. Coefficients and associated p-values genotype-gene model evaluating the association between rs9918437 genotype and gene expression in HER2-enriched subtypes.

Supplementary Figure S1 Figure S1. Generalized pairs plot showing pairwise correlations among genes significantly associated with the rs140068132 genotype.

## Data Availability

Data are in the process of being submitted to the Database of Genotypes and Phenotypes but until then, researchers can reach out to the corresponding author for data access.
