# Supplementary material for: Breast Cancer–Protective Genetic Variant Common in Women of Indigenous American Genetic Ancestry Is a cis-eQTL for RMND1, ARMT1, and CCDC170 in HER2-Enriched Tumor Subtypes
Source: Cancer Res Commun. 2026 Oct 9;6(10):2360–70. doi: 10.1158/2767-9764.CRC-26-0293 (PMC13651766; doi:10.1158/2767-9764.CRC-26-0293)
Supplement: Supplementary file 1 [file crc-26-0293_supplementary_figure_s1_suppst1.docx]

**Supplementary figure 1:** Generalized pairs plot showing pairwise correlations among genes significantly associated with the rs140068132 genotype. Values labeled *Corr* represent Pearson’s correlation coefficients calculated using log10-transformed raw count values, both across all samples and separately within each molecular subtype.
