# Supplementary material for: Breast Cancer–Protective Genetic Variant Common in Women of Indigenous American Genetic Ancestry Is a cis-eQTL for RMND1, ARMT1, and CCDC170 in HER2-Enriched Tumor Subtypes
Source: Cancer Res Commun. 2026 Oct 9;6(10):2360–70. doi: 10.1158/2767-9764.CRC-26-0293 (PMC13651766; doi:10.1158/2767-9764.CRC-26-0293)
Supplement: Supplementary file 2 [file crc-26-0293_supplementary_table_s1_suppst1.docx]

**Supplementary table 1**: Coefficients and associated p-values for the interaction factor in a genotype–subtype interaction model evaluating the association between rs140068132 genotype and gene expression within a 1Mb window from rs140068132. HER2-enriched subtype was defined as the reference group. Age at diagnosis and Indigenous American genetic ancestry were included as covariates. Base Mean: average of the normalized gene count values. log2FC (log2 fold change): estimated effect size. lfcSE (log2FC standard error): standard error estimate for the log2FC. Wald statistic: test statistic calculated as the log2FC divided by the lfcSE. p-value: unadjusted p-value associated with the Wald statistic; p-adjusted: FDR-adjusted p-value for multiple testing across all genes, calculated using the Benjamini–Hochberg procedure. P-adjusted values < 0.05 were considered statistically significant.

| **Gene** | **Base Mean** | **log2FC** | **lfcSE** | **Wald Statistic** | **p-value** | **p-adjusted** |
| --- | --- | --- | --- | --- | --- | --- |
| **Effect of rs140068132 in HER2-enriched (reference)** | | | | | | |
| AKAP12 | 956.33 | 0.72 | 0.25 | 2.83 | 0.005 | 0.144 |
| ARMT1 | 1203.16 | 1.16 | 0.25 | 4.60 | <0.001 | **0.002** |
| CCDC170 | 810.8 | 1.40 | 0.31 | 4.56 | <0.001 | **0.002** |
| ESR1 | 7949.17 | 0.99 | 0.38 | 2.63 | 0.009 | 0.185 |
| MTHFD1L | 1508.49 | -0.35 | 0.17 | -2.03 | 0.043 | 0.377 |
| PLEKHG1 | 1687.87 | -0.27 | 0.18 | -1.51 | 0.130 | 0.572 |
| RMND1 | 1157.56 | 1.01 | 0.24 | 4.22 | <0.001 | **0.005** |
| SYNE1 | 18107.46 | 0.07 | 0.19 | 0.39 | 0.694 | 0.945 |
| ZBTB2 | 659.72 | 0.58 | 0.14 | 4.07 | <0.001 | **0.008** |
| **Effect of rs140068132 in Luminal A compared to HER2-enriched** | | | | | | |
| AKAP12 | 956.33 | -0.68 | 0.37 | -1.88 | 0.061 | 0.540 |
| ARMT1 | 1203.16 | -1.25 | 0.36 | -3.44 | 0.001 | 0.116 |
| CCDC170 | 810.8 | -1.60 | 0.44 | -3.64 | <0.001 | 0.081 |
| ESR1 | 7949.17 | -1.16 | 0.54 | -2.13 | 0.033 | 0.464 |
| MTHFD1L | 1508.49 | 0.46 | 0.25 | 1.86 | 0.063 | 0.542 |
| PLEKHG1 | 1687.87 | 0.37 | 0.25 | 1.45 | 0.147 | 0.680 |
| RMND1 | 1157.56 | -1.01 | 0.34 | -2.92 | 0.004 | 0.227 |
| SYNE1 | 18107.46 | 0.01 | 0.27 | 0.04 | 0.969 | 1.000 |
| ZBTB2 | 659.72 | -0.58 | 0.21 | -2.83 | 0.005 | 0.245 |
| **Effect of rs140068132 in Luminal B compared to HER2-enriched** | | | | | | |
| AKAP12 | 956.33 | -0.71 | 0.33 | -2.15 | 0.032 | 0.38 |
| ARMT1 | 1203.16 | -0.46 | 0.33 | -1.40 | 0.163 | 0.66 |
| CCDC170 | 810.8 | -1.12 | 0.40 | -2.84 | 0.004 | 0.16 |
| ESR1 | 7949.17 | -0.86 | 0.49 | -1.76 | 0.079 | 0.52 |
| MTHFD1L | 1508.49 | 0.12 | 0.22 | 0.52 | 0.600 | 0.92 |
| PLEKHG1 | 1687.87 | 0.26 | 0.23 | 1.12 | 0.261 | 0.75 |
| RMND1 | 1157.56 | -0.61 | 0.31 | -1.97 | 0.049 | 0.44 |
| SYNE1 | 18107.46 | -0.05 | 0.24 | -0.19 | 0.846 | 0.98 |
| ZBTB2 | 659.72 | -0.66 | 0.19 | -3.53 | <0.001 | 0.05 |
| **Effect of rs140068132 in Basal-like compared to HER2-enriched** | | | | | | |
| AKAP12 | 956.33 | -1.1 | 0.4 | -2.8 | 0.005 | 0.224 |
| ARMT1 | 1203.16 | -1.2 | 0.4 | -3.0 | 0.003 | 0.175 |
| CCDC170 | 810.8 | -1.7 | 0.5 | -3.4 | 0.001 | 0.105 |
| ESR1 | 7949.17 | -1.9 | 0.6 | -3.2 | 0.002 | 0.141 |
| MTHFD1L | 1508.49 | 0.8 | 0.3 | 2.9 | 0.004 | 0.203 |
| PLEKHG1 | 1687.87 | 0.2 | 0.3 | 0.7 | 0.506 | 0.940 |
| RMND1 | 1157.56 | -0.9 | 0.4 | -2.4 | 0.014 | 0.351 |
| SYNE1 | 18107.46 | -0.4 | 0.3 | -1.2 | 0.237 | 0.832 |
| ZBTB2 | 659.72 | -0.5 | 0.2 | -2.0 | 0.049 | 0.552 |
