# Supplementary material for: Breast Cancer–Protective Genetic Variant Common in Women of Indigenous American Genetic Ancestry Is a cis-eQTL for RMND1, ARMT1, and CCDC170 in HER2-Enriched Tumor Subtypes
Source: Cancer Res Commun. 2026 Oct 9;6(10):2360–70. doi: 10.1158/2767-9764.CRC-26-0293 (PMC13651766; doi:10.1158/2767-9764.CRC-26-0293)
Supplement: Supplementary file 3 [file crc-26-0293_supplementary_table_s2_suppst2.docx]

**Supplementary table 2:** Coefficients and associated p-values genotype-gene model evaluating the association between rs9918437 genotype and gene expression in HER2-enriched subtypes. A model including rs1468132 and rs9918437 was also include to test independent effects. Age at diagnosis and Indigenous American genetic ancestry were included as covariates. Base Mean: average of the normalized gene count values. log2FC (log2 fold change): estimated effect size. lfcSE (log2FC standard error): standard error estimate for the log2FC. Wald statistic: test statistic calculated as the log2FC divided by the lfcSE. p-value: unadjusted p-value associated with the Wald statistic; p-adjusted: FDR-adjusted p-value for multiple testing across all genes, calculated using the Benjamini–Hochberg procedure. P-adjusted values < 0.05 were considered statistically significant.

| **Model** | **Gene** | **Base Mean** | **log2FC** | **lfcSE** | **Wald Statistic** | **p-value** | **p-adjusted** |
| --- | --- | --- | --- | --- | --- | --- | --- |
| **rs140068132 + rs9918437 + age+ NAM in HER2-enriched tumors** | | | | | | | |
| rs140068132 | AKAP12 | 944.52 | 0.69 | 0.27 | 2.58 | 0.010 | 0.201 |
|  | ARMT1 | 628.81 | 0.99 | 0.22 | 4.48 | 7.50E-06 | **0.004** |
|  | CCDC170 | 338.47 | 1.18 | 0.33 | 3.60 | 3.15E-04 | **0.037** |
|  | ESR1 | 1208.40 | 0.68 | 0.47 | 1.46 | 0.145 | 0.583 |
|  | MTHFD1L | 1452.29 | -0.33 | 0.15 | -2.20 | 0.028 | 0.311 |
|  | PLEKHG1 | 1555.99 | -0.25 | 0.19 | -1.31 | 0.191 | 0.639 |
|  | RMND1 | 801.20 | 0.88 | 0.24 | 3.73 | 1.89E-04 | **0.030** |
|  | SYNE1 | 17839.91 | 0.07 | 0.17 | 0.39 | 0.696 | 0.938 |
|  | ZBTB2 | 667.12 | 0.55 | 0.18 | 3.10 | 0.002 | 0.097 |
| rs9918437 | AKAP12 | 944.52 | -0.49 | 0.26 | -1.91 | 0.057 | 0.623 |
|  | ARMT1 | 628.81 | -0.29 | 0.21 | -1.37 | 0.171 | 0.777 |
|  | CCDC170 | 338.47 | 0.12 | 0.32 | 0.40 | 0.692 | 0.984 |
|  | ESR1 | 1208.40 | -1.20 | 0.45 | -2.68 | 0.007 | 0.347 |
|  | MTHFD1L | 1452.29 | 0.06 | 0.15 | 0.40 | 0.686 | 0.982 |
|  | PLEKHG1 | 1555.99 | 0.09 | 0.18 | 0.47 | 0.639 | 0.976 |
|  | RMND1 | 801.20 | -0.24 | 0.23 | -1.05 | 0.292 | 0.856 |
|  | SYNE1 | 17839.91 | -0.04 | 0.17 | -0.24 | 0.812 | 0.999 |
|  | ZBTB2 | 667.12 | -0.01 | 0.17 | -0.07 | 0.940 | 0.999 |
| **rs9918437 + age+ NAM in HER2-enriched tumors** | | | | | | | |
| rs9918437 | AKAP12 | 944.522 | -0.581 | 0.265 | -2.195 | 0.028 | 0.513 |
|  | ARMT1 | 628.808 | -0.428 | 0.239 | -1.791 | 0.073 | 0.636 |
|  | CCDC170 | 338.469 | -0.231 | 0.336 | -0.687 | 0.492 | 0.939 |
|  | ESR1 | 1208.399 | -1.245 | 0.451 | -2.763 | 0.006 | 0.307 |
|  | MTHFD1L | 1452.295 | 0.068 | 0.149 | 0.454 | 0.650 | 0.976 |
|  | PLEKHG1 | 1555.991 | 0.093 | 0.185 | 0.502 | 0.615 | 0.968 |
|  | RMND1 | 801.198 | -0.391 | 0.246 | -1.593 | 0.111 | 0.700 |
|  | SYNE1 | 17839.909 | -0.046 | 0.165 | -0.276 | 0.782 | 0.993 |
|  | ZBTB2 | 667.119 | -0.086 | 0.179 | -0.479 | 0.632 | 0.970 |
